# Cancer Surveillance in Healthy Carriers of Germline Pathogenic Variants in *BRCA1/2*: A Review of Secondary Prevention Guidelines

**DOI:** 10.1155/2020/9873954

**Published:** 2020-06-20

**Authors:** Boudewijn Dullens, Robin de Putter, Matteo Lambertini, Angela Toss, Sileny Han, Els Van Nieuwenhuysen, Toon Van Gorp, Adriaan Vanderstichele, Chantal Van Ongeval, Machteld Keupers, Renate Prevos, Valerie Celis, Jeroen Dekervel, Wouter Everaerts, Hans Wildiers, Ines Nevelsteen, Patrick Neven, Dirk Timmerman, Ann Smeets, Ellen Denayer, Griet Van Buggenhout, Eric Legius, Kevin Punie

**Affiliations:** ^1^Department of General Medical Oncology, University Hospitals Leuven, Leuven, Belgium; ^2^Multidisciplinary Breast Centre, UZ-KU Leuven Cancer Institute (LKI), University Hospitals Leuven, Leuven, Belgium; ^3^Department of Medical Genetics, Ghent University Hospital, Ghent, Belgium; ^4^Department of Medical Oncology, U.O.C Clinica di Oncologia Médica, IRCCS Ospedale Policlinico San Martino, Genova, Italy; ^5^Department of Internal Medicine and Medical Specialties (DiMI), School of Medicine, University of Genova, Genova, Italy; ^6^Department of Oncology and Hematology, University Hospital of Modena, Modena, Italy; ^7^Department of Gynecology and Obstetrics, University Hospitals Leuven, Leuven, Belgium; ^8^Department of Radiology, University Hospitals Leuven, Leuven, Belgium; ^9^Digestive Oncology, Department of Gastroenterology, University Hospitals Leuven, Leuven, Belgium; ^10^Department of Urology, University Hospitals Leuven, Leuven, Belgium; ^11^Department of Surgical Oncology, University Hospitals Leuven, Leuven, Belgium; ^12^Department of Human Genetics, KU Leuven, Leuven, Belgium

## Abstract

Germline pathogenic alterations in the breast cancer susceptibility genes 1 (*BRCA1*) and 2 (*BRCA2*) are the most prevalent causes of hereditary breast and ovarian cancer. The increasing trend in proportion of cancer patients undergoing genetic testing, followed by predictive testing in families of new index patients, results in a significant increase of healthy germline *BRCA1/*2 mutation carriers who are at increased risk for breast, ovarian, and other *BRCA*-related cancers. This review aims to give an overview of available screening guidelines for female and male carriers of pathogenic or likely pathogenic germline *BRCA1/2* variants per cancer type, incorporating malignancies that are more or less recently well correlated with *BRCA1/2*. We selected guidelines from national/international organizations and/or professional associations that were published or updated between January 1, 2015, and February 1, 2020. In total, 12 guidelines were included. This review reveals several significant discordances between the different guidelines. Optimal surveillance strategies depend on accurate age-specific cancer risk estimates, which are not reliably available for all *BRCA*-related cancers. Up-to-date national or international consensus guidelines are of utmost importance to harmonize counseling and proposed surveillance strategies for *BRCA1/2* carriers.

## 1. Introduction

Germline pathogenic alterations in the breast cancer susceptibility genes 1 (*BRCA1*) and 2 (*BRCA2*) are the most prevalent causes of hereditary breast and ovarian cancer (HBOC). Family studies and segregation analyses have estimated carrier rates of pathogenic and likely pathogenic *BRCA1* or *BRCA2* alterations in a mixed western population between 1 in 200 and 1 in 1500 persons with most estimates towards the lower end of the range [1, 2]. In some populations like Ashkenazi Jews, founder effects are observed with carrier frequencies up to 1% or more [3]. Better knowledge of the implications of *BRCA* alterations in cancer treatment led to higher awareness among patients and physicians. Together with improved availability of genetic testing, this has led to lower testing thresholds and more germline diagnostic tests, resulting in an increase of cancer patients with known germline pathogenic variants in *BRCA1/2*. Predictive testing in families of new index patients leads to a further increase of healthy carriers with germline alterations correlated with *BRCA1/*2 and other monogenetic causes of HBOC [4].

There are several implications for carriers of (likely) pathogenic variants in *BRCA1*/*2*. Besides the increased cancer risks and the identified prognostic and predictive implications in *BRCA*-related breast, ovarian, pancreatic, and prostate cancers, the autosomal dominant inheritance pattern has important implications for the children and relatives of mutation carriers [5, 6]. Known female and male carriers of pathogenic or likely pathogenic variants who plan to conceive should be counseled about options of prenatal and preimplantation genetic diagnosis [7].

The elevated cancer risks extend beyond breast and ovarian cancer. There is clear evidence for an increased risk for prostate and pancreatic cancer. The risk for other cancers such as stomach, colorectal, and endometrial cancer and melanoma might also be elevated to some extent, and some guidelines give recommendations for these possible associations, while for other reported supposed correlations, none of the guidelines give specific recommendations (e.g., cervical cancer) [8, 9]. There are important uncertainties and differences in strength of evidence and differential effects for *BRCA1* and *BRCA2* with regard to these and other possible additional cancer risks. Lifetime risks have not been reliably estimated for all these correlations (Table 1). Given the burden of cancer risks and surveillance for germline carriers of a hereditary cancer syndrome, appropriate counseling about primary and secondary prevention strategies is a crucial factor in the care for these individuals. Several national and international guidelines and algorithms for surveillance of *BRCA*-related cancers exist. With this review, we aim to give an overview and comparison of available screening guidelines for *BRCA*-related cancers for female and male carriers of pathogenic or likely pathogenic germline *BRCA1/2* variants per cancer type, incorporating malignancies where a correlation with *BRCA1/2* is more or less recently well demonstrated.

## 2. Methods

We selected articles for our review by Medline search and additional web-based search of the national and international organizations and/or professional associations for guidelines that reported recommendations on secondary prevention in female and/or male carriers of pathogenic or likely pathogenic germline *BRCA1/2* variants. Only guidelines published or updated between January 1, 2015, and February 1, 2020, were eligible for inclusion in this review. The review is limited to recommendations available in English, French, or Dutch. Guidelines that did not provide clear information about the starting age of surveillance or about the recommended screening modality were excluded. We retrieved 12 guidelines that met our criteria.

## 3. Cancer Surveillance Guidelines in Germline *BRCA1/2* Mutation Carriers

### 3.1. Breast Cancer

Germline pathogenic variants in *BRCA1/2* are highly penetrant for breast cancer. The incidence of breast cancer in female *BRCA1/2* carriers increases rapidly in early adulthood. The breast cancer risk increases between 30 and 40 years in *BRCA1*, but the higher penetrance of *BRCA2* at later ages has been confirmed reaching an absolute cumulative risk between 60 and 80% at age 80 years for both *BRCA1* and *BRCA2*. The risk of contralateral breast cancer is estimated at 40% for *BRCA1* carriers and 25% for *BRCA2* carriers at 20 years after the first breast cancer diagnosis [10].

The high lifetime risk of breast cancer in female *BRCA* carriers makes the discussion of primary prevention strategies (lifestyle modifications, chemoprevention, and risk-reducing surgery) important. Regarding chemoprevention, only limited data exist on the preventive benefit of tamoxifen in *BRCA1/2* mutation carriers. In addition, there is some concern about the safety of tamoxifen regarding endometrial cancer risk. Moreover, there is discordance as to whether *BRCA1* carriers, who are more prone to estrogen receptor negative breast cancer, benefit as much from this chemoprevention approach as *BRCA2* carriers [22, 23]. Several trials investigating new chemoprevention approaches in *BRCA* carriers are ongoing [24].

Risk-reducing mastectomy (RRM) has been shown to be a very effective breast cancer primary prevention option [25, 26]. Breast cancer after RRM in *BRCA* carriers has been reported, but the absolute risk is very low and none of the guidelines propose imaging surveillance after RRM [25, 27]. A cohort study has shown improved overall and breast cancer-specific mortality rates in *BRCA1* mutation carriers, while for *BRCA2*, survival rates were not significantly different after a median follow-up for 10.3 years [28]. In clinical practice, for the vast majority of women opting for RRM, mortality reduction is not the dominant driver in the decision process [29]. The option of RRM should be discussed with female carriers of (likely) pathogenic germline mutations in *BRCA1/2*. However, risk-reducing surgery should never be recommended as the only option to address the high breast cancer risk, and the advantages and disadvantages of this option and other primary or secondary prevention strategies should be extensively discussed.

Although male breast cancer is a rare disease in the general population, with a lifetime risk of 0.1% accounting for less than 1% of all cancers in men and about 1% of all breast cancers, the cumulative incidence is significantly increased in male *BRCA1/2* carriers and is estimated at 1% in *BRCA1* carriers and 7-8% in *BRCA2* carriers [12, 13, 30].

**Figure 1:**
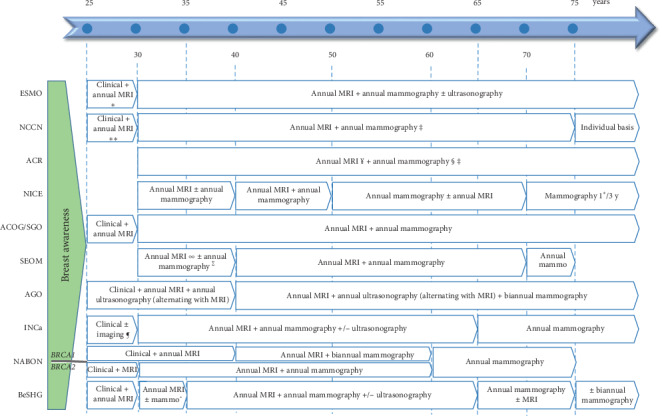
Schematic overview of surveillance guidelines for breast cancer in asymptomatic female carriers of (likely) pathogenic *BRCA1*/*2* variants. MRI: magnetic resonance imaging; ESMO: European Society for Medical Oncology; NCCN: National Comprehensive Cancer Network; ACR: American College of Radiology; NICE: National Institute for Health and Care Excellence; ACOG: American College of Obstetricians and Gynecologists; SGO: Society of Gynecologic Oncology; SEOM: Sociedad Espanola de Oncologia Médica; AGO: Arbeitsgemeinschaft Gynäkologische Onkologie; INCa: Institut National du Cancer; NABON: Nationaal Borstkanker Overleg Nederland; BeSHG: Belgian Society of Human Genetics. ^*∗*^Or starting 10 years earlier than youngest breast cancer diagnosis in the family. ^*∗∗*^Or individualized based on family history if a breast cancer diagnosis is present before age 30. ^¥^Or starting 5–10 years earlier than the youngest breast cancer diagnosis in the family. ^§^Starting 10 years before the youngest breast cancer diagnosis in the family, but not before 30. ^‡^Considering breast tomography. ^∞^Or starting earlier if there is a family history of breast cancer before 30 years. ^∑^Discussing delaying mammography until 40 years with *BRCA1* carriers who undergo annual MRI screening. ^¶^Considering imaging in case of early breast cancer diagnosis in the family. ˜Mammography at age 30, annual mammography from 30 onwards in case of microcalcifications.

Breast cancer screening in germline mutation carriers is correlated with an increased rate of stage 0 or stage 1 breast cancer, and there is limited data about survival benefit [31, 32]. There are several guidelines and recommendations for breast cancer surveillance in germline *BRCA* mutation carriers. A schematic overview of guidelines for female carriers is shown in Figure 1. The majority of guidelines address screening approaches for female and male carriers and discuss recommendations on breast awareness, clinical examination, mammography, and magnetic resonance imaging (MRI), but guidance on the use of digital breast tomosynthesis (DBT) and ultrasound is often not specified. There is concern that the exposure to diagnostic radiation at young age may be associated with an increased risk of breast cancer in *BRCA* carriers [33]. Moreover, the decision as to whether or not to undergo a RRM is often not made at the recommended starting age for breast cancer screening. Therefore, the starting age of mammography in female carriers is an important aspect of the surveillance guidelines. Some guidelines advise annual screening procedures, while the concern about interval cancers in these high-risk patients leads to semiannual alternating schedules in other recommendations [31]. The option of DBT is mentioned in some guidelines based on the superior sensitivity and specificity compared to standard mammography; however, there is no data on the use in *BRCA* mutation carriers who undergo MRI screening [34]. In a recent study among 1444 average-risk women aged 40–70 with heterogeneously dense or extremely dense breast, the invasive cancer detection rate was significantly higher for MRI compared to DBT, and no invasive cancer was identified by DBT alone [35].

#### 3.1.1. European Society for Medical Oncology Guidelines

The clinical practice guidelines for cancer prevention and screening in *BRCA* mutation carriers from the European Society for Medical Oncology (ESMO) were published in 2016 [36]. For female carriers of pathogenic *BRCA* variants, breast awareness and clinical breast examination are recommended every 6–12 months from the age of 25 or 10 years before the youngest breast cancer diagnosis in the family, whichever occurs first. Annual MRI is recommended from the age of 25, with the addition of annual mammography from the age of 30. The decision to introduce mammography before the age of 40 should take into consideration the increased breast density at younger ages and the availability of annual screening MRI. In women ≤30 years, breast ultrasound can be considered in case MRI is unavailable. Ultrasound can also be considered in addition to mammography at all ages and as an alternative when MRI is not available. Upper age limit or other conditions where screening should be discontinued are not described for female carriers. After RRM, routine surveillance is not recommended but should be considered in patients who have undergone nipple-sparing mastectomy. Male carriers should be advised to undergo annual clinical breast examination by a physician from age 30 onwards. Routine annual breast imaging among male carriers is not recommended.

#### 3.1.2. National Comprehensive Cancer Network Guidelines

The last version from the clinical practice guidelines in genetic high-risk assessment for breast, ovarian, and pancreatic cancer of the National Comprehensive Cancer Network (NCCN) dates from December 2019 [37]. With regard to breast cancer surveillance in women, breast awareness is recommended starting at age 18 and clinical breast exam every 6–12 months from age 25. Between age 25 and 29, annual breast MRI with contrast is recommended. Starting age should be individualized based on family history if a breast cancer was diagnosed in a relative before age 30. When MRI is unavailable, annual mammogram with consideration of DBT is recommended. Between ages 30 and75, both annual MRI with contrast and annual mammogram with consideration of DBT are recommended. In carriers >75 years, management should be considered on an individual basis. Criteria for high-quality breast MRI include availability of experienced breast MRI radiologists, a dedicated breast coil, the ability to perform MRI-guided biopsies, and regional availability. Breast MRI is preferably performed on days 7–15 of a menstrual cycle in premenopausal women.

Male carriers of (likely) pathogenic variants in *BRCA* are recommended to undergo annual clinical breast examination and undergo training in self-examination with monthly practice starting from age 35 onwards. Regularly scheduled mammography is not recommended in male *BRCA* carriers.

#### 3.1.3. American College of Radiology

The publication of the Appropriateness Criteria® for breast cancer screening from the American College of Radiology (ACR) dates from 2017 [34]. Recommendations are limited to the radiological imaging procedures, and guidelines for breast cancer screening in women with a *BRCA* gene mutation are similar to the recommendations for women with a history of chest irradiation between 10 and 30 years of age and women with ≥20% lifetime breast cancer risk. Annual mammography is recommended starting 10 years earlier than the affected relative at the time of diagnosis, but not before 30 years. The superior sensitivity and specificity of DBT over planar mammography are described, and the advantages seem to be most pronounced in women with higher breast density, in women under age 50, and in carriers with spiculated masses and asymmetries. Since in the majority of situations standard two-dimensional images are obtained in addition to the DBT images, the radiation dose is increased compared to standard mammography. However, virtual planar images created from the tomographic data set could replace the need for a 2D correlative view in the near future. Surveillance with annual breast MRI (with and without contrast) is recommended in addition to mammography. For the starting age of MRI screening in *BRCA* carriers, ACR refers to the American Cancer Society Guidelines for breast screening with MRI as an adjunct to mammography. The recommended starting age is 30 years for the majority of women, or 5 to 10 years before the earliest breast cancer diagnosis in the family. The starting age should be based on shared decision making, considering individual preferences and circumstances. Screening with breast MRI should be continued as long as the woman is in good health.

#### 3.1.4. National Institute for Health and Care Excellence Guidelines

The clinical guidelines on familial breast cancer by the National Institute for Health and Care Excellence (NICE) were originally published in 2013, but the online version was verified as up-to-date in November 2019 [38]. All carriers should be informed about breast awareness. Annual mammography should be considered in female carriers aged 30–39 and recommended aged 40–69, while patients ≥70 years should be offered mammography every three years as part of the population screening program. Mammographic surveillance should never be offered for patients <30 years.

Annual MRI surveillance should be offered to female carriers aged 30–49 years and can be considered between 50 and 69 years in case of dense breast pattern but should not be offered to *BRCA* carriers <30 years.

The NICE guidelines state that ultrasound surveillance should not be routinely offered but could be considered when MRI is not possible or when results of mammography or MRI are difficult to interpret. No recommendations are made for male carriers.

The guidelines on breast cancer screening from the London Cancer Alliance (published in 2013 and updated in 2016, [39]) and the Institute of Cancer Research protocol for *BRCA* mutation carriers (2015, [40]) are concordant with the NICE guidelines. The latter specifies that no breast surveillance is recommended for male carriers.

#### 3.1.5. American College of Obstetricians and Gynecologists/Society of Gynecologic Oncology

The HBOC clinical management guidelines from the committee on practice bulletins from the American College of Obstetricians and Gynecologists (ACOG) and the committee on genetics from the Society of Gynecologic Oncology (SGO) were last reviewed in 2017 [41]. For woman aged 25–29, recommended surveillance consists of clinical breast examination every 6–12 months in combination with annual radiographic screening (preferably MRI with contrast). For women ≥30 years, annual mammography and annual MRI with contrast are recommended, often alternating every 6 months. There are no specific statements regarding the use of ultrasonography, or about age limits or male carriers, in these guidelines.

#### 3.1.6. Spanish Society of Medical Oncology

The clinical guidelines in HBOC of the hereditary cancer working group from the Spanish Society of Medical Oncology (Sociedad Espanola de Oncologia Médica, SEOM) were revised in 2019 [42]. Annual breast MRI should be proposed between 30 and 70 years, or earlier in case of family history of breast cancer before 30 years. Addition of annual mammogram should be considered from 30 years onwards and recommended between 40 and 75 years. Delaying mammography until 40 years should be discussed for *BRCA1* carriers who undergo annual MRI screening.

When MRI is unavailable, screening with mammography and ultrasound is advised between 30 and 75 years. For male *BRCA* carriers, the SEOM guidelines advise that screening mammography should be considered only in the presence of gynecomastia.

#### 3.1.7. German Society for Gynecological Oncology

The proposed surveillance program of the German Society for Gynecological Oncology (Arbeitsgemeinschaft Gynäkologische Onkologie, AGO) is available in the latest version of the AGO breast guidelines which were last revised in 2019 [43]. Clinical breast examination is recommended semiannually for female carriers from age 25 onwards. Starting age for annual breast MRI is 25 years. Annual ultrasonography is recommended in interval between the MRI examinations from age 25 onwards. Biannual mammography is recommended starting at age 40. In upper age limit, other conditions where screening should be discontinued and recommendations for male carriers are not described.

#### 3.1.8. French National Cancer Institute Guidelines

The guidelines on early breast and ovarian cancer detection and risk-reducing strategies for female *BRCA* carriers from the French National Cancer Institute (Institut National du Cancer, INCa) were published in 2017 [44]. In female carriers <30 years of age, annual clinical breast exam is recommended, with the addition of imaging only in case of early familial antecedents. Between age 30 and 65, annual synchronous MRI and mammogram are recommended with the addition of ultrasonography on indication, six-monthly alternating with a clinical breast exam. Specific guidance on imaging technique (e.g., single oblique incidence in conjunction with breast MRI) and radiologist requirements are described. For female carriers above age 65, annual mammography (double incidence) is recommended. Regarding the upper age limit, comorbidities and life expectancy have to be considered.

#### 3.1.9. National Breast Cancer Council Netherlands

The breast cancer surveillance guidance for *BRCA* mutation carriers from the Dutch national breast cancer guidelines (Nationaal Borstkanker Overleg Nederland, NABON) were last revised in 2017 [45]. Annual clinical breast examination is recommended between 25 and 75 years. Interestingly, regarding breast imaging guidelines, a differentiation between *BRCA1* and *BRCA2* is made. For *BRCA1* carriers, only annual breast MRI is advised between 25 and 40 years. Between age 40 and 60, annual MRI and biannual mammogram is recommended. For *BRCA2* carriers, annual breast MRI is recommended from age 25 onwards, with the addition of annual mammogram starting at age 30. Between age 60 and age 75 annual mammogram is recommended, where in case of high breast density annual imaging with alternating MRI and mammogram should be considered, both in *BRCA1* and *BRCA2* carriers.

#### 3.1.10. Belgian Society of Human Genetics

The Belgian guidelines for managing hereditary breast and ovarian cancer were developed in 2019 within the working group oncogenetics from the College of Genetics and Rare disease and the Belgian Society of Human Genetics (BeSHG) and are endorsed by the hereditary cancer task force of the Belgian society of Medical Oncology (BSMO) [46]. For female *BRCA* carriers, clinical breast examination is recommended every 6 months from age 25 onwards. Between age 25–35, annual breast MRI is advised. At age 30 a first baseline mammogram is recommended. In case microcalcifications are present as a possible reflection of in situ carcinoma, yearly mammogram (+/− ultrasound when indicated by the radiologist) should be recommended from age 30 onwards in situations where no treatment is indicated yet, whereas in the absence of these findings annual mammogram can be considered from 30 onwards, but is only routinely recommended from age 35. Between 35 and 65 years, both breast MRI and mammogram (+/− ultrasound) are recommended, alternating every 6 months. Between age 65 and 75, annual mammography is recommended, and MRI should only be considered in case of residual dense breast tissue or other findings on breast imaging where added value of MRI could be expected. For women >75 years, a biannual mammogram should be considered. With regard to male breast cancer, routine screening is not recommended for *BRCA1*, while for *BRCA2* annual clinical exam can be considered starting from age 40 onwards.

### 3.2. Ovarian, Fallopian Tube, and Primary Peritoneal Cancer

Carriers of a pathogenic *BRCA* mutation are at high risk for epithelial ovarian, fallopian tube and primary peritoneal cancer, with a cumulative risk at 80 years of 44% for *BRCA1* and 17% for *BRCA2* [10, 47]. Ovarian cancer incidence increases slowly from approximately 35 years onwards in patients with *BRCA1*-and from around 50 years onwards in *BRCA2*-mutations. In contrast to breast cancer where both prophylactic mastectomy and medical surveillance are reasonable, outcomes of epithelial ovarian cancer are poor and there are major limitations regarding early detection. Risk-reducing salpingo-oophorectomy (RRSO) provides an important reduction in ovarian and breast cancer risks and related mortality; however, the latter is less clearly demonstrated for *BRCA2* [48–50]. Therefore, all female carriers with (likely) pathogenic *BRCA* variants should be recommended to undergo risk-reducing surgery of the fallopian tubes and ovaries after completion of childbearing [37]. With regard to the timing of surgery, quality of life and age-adjusted ovarian cancer risk should be considered. In *BRCA1* carriers, RRSO is usually advised between the age of 35 to 40, after completion of childbearing. Because later onset of disease in BRCA2 mutation carriers, RRSO can be advised between the age of 40–50, however some guidelines still use the 35 lower age limit for RRSO recommendation for *BRCA2* [37, 46, 51]. Although there is some evidence regarding the safety of interval salpingectomy (with retention of the ovaries) as initial procedure with the goal to decline or delay menopause initiation, more data are needed before this can be routinely recommended. Clinical trials investigating the safety of this procedure are currently ongoing (e.g., NCT02321228) [52]. Due to this strong recommendation for risk-reducing surgery, ovarian cancer surveillance only is applicable in patients who refuse or have not yet undergone RRSO. Primary peritoneal carcinoma after RRSO has been reported mainly in *BRCA1* carriers but remains rare. Moreover this stays a controversial entity since this could possibly reflect a metastatic lesion arising from serous tubal intraepithelial carcinoma (STIC), which is a precursor lesion of high-grade serous ovarian cancer [53, 54]. Therefore, the risk of primary peritoneal carcinoma in *BRCA* carriers is not discussed in the majority of secondary prevention guidelines. Adequate pathological examination of RRSO specimens by the ‘standardized sectioning and extensively examining the fimbriated end' protocol (SEE-FIM) is necessary in order to detect the presence of precancerous lesions in the fallopian tube, e.g., serous tubal intraepithelial carcinomas [55]. These lesions warrant further staging, as they were correlated with metastatic potential in sporadic ovarian cancer [54].

#### 3.2.1. European Society for Medical Oncology Guidelines

The clinical practice guidelines for cancer prevention and screening in *BRCA* mutation carriers from ESMO emphasize the technical limitations for early detection of ovarian cancer and that there are no data proving that screening for ovarian cancer in *BRCA* carriers reduces mortality [36]. There are some promising results with serial CA125 screening, but sufficient data are unavailable [56]. Before RRSO, six-monthly transvaginal ultrasound and serial measures of serum CA125 could be considered from the age of 30. The limited data on this approach should be communicated with the patient. After RRSO, surveillance for the residual risk of peritoneal carcinoma is not recommended.

#### 3.2.2. National Comprehensive Cancer Network Guidelines

The NCCN clinical practice guidelines in genetic high-risk assessment for breast, ovarian and pancreatic cancer state that transvaginal ultrasound combined with serum CA125 measures for ovarian cancer screening may be considered in *BRCA* mutation carriers who have not underwent elective RRSO starting at age 30 to 35. The benefit of this screening is uncertain.

#### 3.2.3. American College of Obstetricians and Gynecologists/Society of Gynecologic Oncology

The HBOC clinical management guidelines from the ACOG/SGO do not generally recommend routine ovarian cancer screening with measurement of serum CA125 or transvaginal ultrasonography [41]. These screening procedures have not proven to decrease mortality rate or increase survival rate associated with ovarian cancer-specific mortality. Transvaginal ultrasonography or CA125 measures are reasonable options for short-term surveillance in women at high risk of ovarian cancer, starting at age 30–35 years and continuing until they opt for RRSO.

#### 3.2.4. American College of Radiology

The publication of the Appropriateness Criteria® for ovarian cancer screening from the American College of Radiology (ACR) dates from 2017 [57]. They state that transvaginal, transabdominal and color Doppler of the ovaries may be appropriate in premenopausal and postmenopausal *BRCA* carriers, and specify that other imaging techniques are usually not appropriate.

#### 3.2.5. Spanish Society of Medical Oncology

The SEOM clinical guidelines in HBOC advise considering six-monthly transvaginal ultrasound and CA125 measures from the age of 30 in *BRCA1*/2 mutation carriers until the age of RRSO, as well as for those who have not elected RRSO [42].

#### 3.2.6. French National Cancer Institute Guidelines

The French guidelines by INCa recommend annual clinical pelvic examination as screening for ovarian cancer in *BRCA1* and *BRCA2* mutation carriers [44]. Starting age is not specified.

#### 3.2.7. National Breast and Ovarian Cancer Council Netherlands

The breast cancer surveillance guidance for *BRCA* mutation carriers from the NABON specifies that screening for ovarian cancer in *BRCA1* and *BRCA2* mutation carriers is not recommended [45].

The familial and hereditary ovarian cancer guidelines from the Dutch cancer center (Integraal Kankercentrum Nederland, IKNL) advise counseling carriers on the absence of data that supports effectivity of ovarian screening and recommend not offering ovarian screening to *BRCA* carriers [58].

#### 3.2.8. Belgian Society of Human Genetics

The Belgian guidelines for managing HBOC do not recommend screening for ovarian cancer in *BRCA1* or *BRCA2* mutation carriers [46]. A tailored screening program could be offered from age 40, when the patient refuses RRSO.

#### 3.2.9. Institute of Cancer Research

While the NICE guidelines do not mention the option of ovarian cancer screening, the ICR BRCA mutation carrier guidelines specify that ovarian surveillance is not recommended [40].

### 3.3. Pancreatic Cancer

The risk of pancreatic adenocarcinoma is increased in *BRCA2* mutation carriers, while data for *BRCA1* are conflicting [9, 14, 59]. Screening in high-risk patients, like *BRCA* mutation carriers with familial antecedents, might be beneficial given the high mortality rate of pancreatic adenocarcinoma. Data suggest that screening is able to detect earlier stages of pancreatic cancer that are still curable, in comparison to people who are diagnosed with symptomatic disease [60]. Also in pancreatic cancer, *BRCA* pathogenic variants have therapeutic implications [6]. Given the well documented correlation between smoking and pancreatic adenocarcinoma, additional counseling for smoking cessation in this regard seems to be an important primary prevention strategy in *BRCA* mutation carriers [61, 62].

#### 3.3.1. European Society for Medical Oncology Guidelines

The ESMO clinical practice guidelines for cancer prevention and screening in *BRCA* mutation carriers state that annual screening for pancreatic cancer may be considered in *BRCA2* mutation carriers [36]. People should be informed about the very limited available data for this approach. There is no consensus about when screening should be initiated, but it is reasonable to start at age 50 or 10 years before the earliest diagnosed case of pancreatic cancer in the family. Screening consists of endoscopic ultrasound (EUS) or MRI/magnetic resonance cholangiopancreatography (MRCP). Trials evaluating the efficacy of screening techniques for pancreatic cancers should be strongly encouraged for *BRCA* carriers.

#### 3.3.2. National Comprehensive Cancer Network Guidelines

The NCCN clinical practice guideline in genetic high-risk assessment for breast, ovarian, and pancreatic cancer does not recommend pancreatic cancer screening for *BRCA1* and *BRCA2* mutation carriers in the absence of a close family history of exocrine pancreatic cancer [37]. Pancreatic cancer screening can be considered for individuals with exocrine pancreatic cancer in one or more first- or second-degree relatives from the same side of the family as the identified pathogenic/likely pathogenic *BRCA1/2* mutation. Screening starts at the age of 50, or 10 years younger than the earliest exocrine pancreatic cancer diagnosis in the family. Screening recommendations include annual contrast-enhanced MRI/MRCP and/or EUS, with consideration of shorter screening interval when worrisome abnormalities are found. The majority of small cystic lesions found on screening will not warrant a biopsy or surgical resection. Before attending screening, people should be informed about the potential limitations to screening, including cost, high incidence of pancreatic abnormalities, and uncertainties about the potential benefits. It is recommended that pancreatic cancer screening should be performed in experienced high-volume centers under research conditions.

#### 3.3.3. Spanish Society of Medical Oncology

The SEOM clinical guidelines in HBOC propose to consider pancreatic cancer surveillance with EUS and MRI in carriers with FDG with pancreatic cancer from the age of 50, or 10 years before the youngest diagnosis in the family [42].

#### 3.3.4. Belgian Society of Human Genetics

The BeSHG guidelines propose to discuss the arguments in favor of and against pancreatic cancer screening with *BRCA1* carriers if they have ≥1 first-degree relative with pancreatic cancer and with *BRCA2* carriers if they have ≥1 first-degree or ≥2 second-degree relatives with pancreatic cancer [46]. This should preferably be performed in the context of a clinical trial. Regarding the starting age and screening modality, they refer to the recommendation from the International Cancer of the Pancreas Screening Consortium [59]. Recommended starting age for *BRCA* carriers with familial antecedents as described above is 50 years, unless there is a first-degree relative with pancreatic cancer onset <50 years. Surveillance for pancreatic cancer should consist of MRI/MRCP and an EUS combined with fasting blood glucose and/or HbA1c. Annual blood sugar tests and imaging are recommended; however, there is no consensus as to whether and how to alternate MRI/MRCP and EUS. Serum CA 19-9 is not routinely recommended.

### 3.4. Prostate Cancer

The link between an elevated risk of prostate cancer and germline *BRCA* pathogenic variants has been well established, with the strongest association for *BRCA2* [63, 64]. Prostate cancer in germline *BRCA2* carriers appears to occur at an earlier age; has a more aggressive phenotype, a higher risk of nodal involvement, and distant metastasis; and is associated with a poor survival outcome in comparison to noncarriers. In advanced castration-resistant prostate cancer, *BRCA* status can have therapeutic implications regarding the use of platinum agents and PARP inhibitors [65, 66].

#### 3.4.1. European Society for Medical Oncology Guidelines

The ESMO clinical practice guidelines state that annual screening for prostate cancer may be considered from age 40 onwards, particularly for *BRCA2* mutation carriers [36]. The optimal duration of screening is not specified but should be tailored to an individual's family history of prostate cancer.

#### 3.4.2. National Comprehensive Cancer Network Guidelines

The NCCN clinical practice guidelines in genetic high-risk assessment for breast, ovarian, and pancreatic cancer refer to the NCCN prostate cancer early detection guidelines for prostate cancer screening in *BRCA* carriers [37, 67]. Prostate cancer screening in *BRCA2* mutation carriers is recommended starting at the age of 40, whereas in *BRCA1* mutation, carriers screening should be considered from the age of 40 onwards. Shared decision making is recommended. In men older than 75 years, prostate cancer screening should be considered in selected patients only. The NCCN prostate cancer early detection guidelines specify yearly screening for PSA. Digital rectal examination (DRE) should not be used as a stand-alone test but may be considered as baseline test and as follow-up exam as it may identify high-grade cancers associated with low serum PSA values. It should be performed in carriers with an elevated serum PSA. Referral for biopsy should be considered if DRE is very suspicious.

#### 3.4.3. Spanish Society of Medical Oncology

The hereditary cancer working group from SEOM recommends prostate cancer screening with annual serum PSA measurements in male *BRCA2* carriers starting at age 40, while this screening approach can also be offered to *BRCA1* carriers [42].

#### 3.4.4. Belgian Society of Human Genetics

The Belgian Society of Human Genetics recommends annual prostate cancer screening with serum PSA and DRE for male *BRCA1* and *BRCA2* mutation carriers from the age of 40 onwards [46].

### 3.5. Colorectal and Gastric Cancer

Data about a possible relationship between gastric and colorectal cancer (CRC) and germline *BRCA* pathogenic variants are conflicting. A large prospective study on 7015 women with *BRCA* alterations showed a significant increased risk for CRC in women younger than 50 years with a *BRCA1* mutation, but not in older *BRCA1* carriers or in *BRCA2* carriers [68, 69]. A systematic review and meta-analysis confirmed the differential effect between *BRCA1* and *BRCA2* (odds ratio [OR] 1.49 [95% CI 1.19–1.85] for *BRCA1*, not significant [OR 1.1; 95% CI 0.77–1.58] for *BRCA2*), but could not validate the age effect [19]. Regarding a possible relationship with gastric cancer, there is only weak evidence for a correlation with germline *BRCA* mutations and gastric cancer; anecdotal findings have not been confirmed in larger series [8, 70, 71]. These recent findings warrant increased attention to familial CRC and possibly gastric cancer antecedents and the need for individualized surveillance in *BRCA* carriers. The majority of guidelines do not mention the possible increased risk for digestive tract cancer.

#### 3.5.1. European Society for Medical Oncology Guidelines

The ESMO clinical practice guidelines state that the association between *BRCA* mutation carriers and an elevated risk of colorectal and gastric cancer is weak [36]. Therefore, screening is generally not indicated. Recommendations should be tailored to an individual's familial history.

#### 3.5.2. Spanish Society of Medical Oncology

The clinical guidelines in HBOC of the hereditary cancer working group from SEOM point towards the controversial results on the association of *BRCA1/2-mutations* and colorectal cancer and towards the possible differences between *BRCA1* and *BRCA2,* but latest version of these guidelines does not mention colorectal cancer surveillance, while specific recommendations for *BRCA1* were reported in the previous version [42, 51].

#### 3.5.3. Belgian Society of Human Genetics

The BeSHG guidelines for managing HBOC indicate that *BRCA1* mutation carriers have an increased risk of early-onset colorectal cancer (diagnosis <50 years), but the increase is small. Screening for colorectal cancer is not recommended for this elevated risk besides the national population screening program independent of *BRCA* status which offers biannual fecal blood test between 50 and 74 years of age in absence of familial history [46]. Also here, a possible correlation with elevated gastric cancer risk is not mentioned.

### 3.6. Endometrial Cancer

Some data suggest a slightly increased risk of endometrial cancer in *BRCA* carriers, with more evidence for a correlation with *BRCA1* and then with *BRCA2*; however, the risk is not clearly defined. Several reports showed that tamoxifen use for previous breast cancer is an important confounding factor in the earlier observed correlations between endometrial cancer and germline *BRCA* mutations [72, 73]. A prospective cohort study analyzing the risk of endometrial cancer after RRSO in 1083 *BRCA* carriers showed no significant increase of endometrial cancer overall, but a higher than expected risk of serous endometrial carcinoma in *BRCA1* mutation carriers (however, only 4 cases were described in 453 *BRCA1* carriers after a median follow-up of 5.1 years), while the risk for endometrioid endometrial cancer or uterine sarcoma was not increased in this study [20]. Another cohort study on 828 carriers could not confirm the correlation with serous endometrial cancer. Overall, no significant correlation with endometrial cancer was demonstrated, but there was a possible trend for the endometrioid subtype [74]. Based on these findings, some guidelines advise discussing these risk uncertainties and the risks and benefits of concurrent hysterectomy at the time of RRSO in female *BRCA1* carriers [37]. However, the majority of guidelines do not recommend considering hysterectomy for the presumed increased risk of endometrial cancer. In female *BRCA* carriers who have opted for breast surveillance instead of risk-reducing mastectomy, there is more data on safety with regard to breast cancer risk of estrogen-only hormonal substitution compared to combined estrogen-progesterone substitution after RRSO [75]. With regard to endometrial cancer risk, however, estrogen-only substitution is not a safe option when no hysterectomy has been performed, making this an additional factor to be considered in these discussions [76].

#### 3.6.1. European Society for Medical Oncology Guidelines

The ESMO clinical practice guidelines for cancer prevention and screening in *BRCA* mutation carriers report that the association between *BRCA1*/*BRCA2* mutations and an elevated risk of endometrial cancer remains weak [36]. They conclude that screening for and prevention of endometrial cancer are generally not indicated. Recommendations should be tailored to an individual's familial history.

#### 3.6.2. National Comprehensive Cancer Network Guidelines

The NCCN guidelines state that there is limited data suggesting there might be a slightly increased risk of serous endometrial cancer among women with a *BRCA1* pathogenic or likely pathogenic variant [37]. The clinical significance is unclear. There is no guidance with regard to screening or prevention. Further evaluation of the risk of serous endometrial cancer in the *BRCA* population needs to be undertaken.

#### 3.6.3. Belgian Society of Human Genetics

Surveillance and prevention of endometrial cancer in *BRCA1* mutation carriers are not advised by the Belgian Society of Human Genetics, because the cumulative risk of serous endometrial cancer is less than 5% at 70 years of age. The risk in *BRCA2* mutation carriers is described as equal to a population without germline *BRCA* pathogenic variants.

### 3.7. Melanoma

Literature suggests a possible association between germline *BRCA2* pathogenic variants and an elevated risk for melanoma. This possible link has been suggested for both cutaneous and ocular melanoma in *BRCA2*, but data are conflicting and mainly based on small studies at risk for sampling bias [9, 15, 21, 77]. Overall there seems to be insufficient evidence for a clear correlation between skin and uveal melanoma and germline *BRCA* pathogenic variants. However, increased awareness of familial history and preventive measurements in *BRCA* carriers seems reasonable.

#### 3.7.1. European Society for Medical Oncology Guidelines

The ESMO guidelines demonstrate that there is no evidence-based data with regard to screening for melanoma [36]. They advise considering annual skin and eye examination as screening for melanoma in all *BRCA2* carriers. Screening should be tailored to the individual's family history.

#### 3.7.2. National Comprehensive Cancer Network Guidelines

The NCCN guidelines of genetic high-risk assessment for breast, ovarian, and pancreatic cancer state that no specific screening guidelines exist for melanoma, but general melanoma risk management with education regarding clinical signs, minimizing UV exposure, and annual full-body skin examination with the addition of an eye exam should be considered for both *BRCA1* and *BRCA2* mutation carriers with a pathogenic or likely pathogenic mutation [37]. An individualized screening approach based on personal and family history of cancer may be provided.

#### 3.7.3. Spanish Society of Medical Oncology

In the SEOM clinical guidelines in HBOC, screening for melanoma with a skin and eye examination should be considered according to personal and familial risk factors [42]. It is not specified if this applies to *BRCA1* and/or *BRCA2* mutation carriers.

## 4. Discussion

This review demonstrates that there are major differences in national and international guidelines on early detection of and screening for *BRCA*-related cancers in *BRCA* carriers. These differences are triggered by temporal evolution in risk assessments, discordances in literature and interpretation, assessment of the advantages and disadvantages of screening, cost-benefit analyses, and absence of high levels of evidence. As the case for cancer screening in the general population, different thresholds and risk/benefit analyses are used by different societies publishing guidelines for HBOC. More harmonized guidelines could be relevant from a clinical perspective, but this is hard to implement at a global level for the reasons stated above. However, harmonization efforts by translation of international guidelines into the local context in regional or national guidelines can be of high value to avoid differences in counseling and risk management advice.

In general, guidelines are more concordant for *BRCA*-related cancers in situations where the age-specific risks for this cancer type are more extensively studied, while there is more discordance in other *BRCA*-related cancers. However, also in breast cancer, there are differences in screening modalities, thresholds, and frequency and duration of screening. The majority of guidelines recommend starting imaging surveillance in female carriers from age 25 onwards and also consider screening for all untested first-degree relatives of *BRCA* carriers [34, 36, 37]. Only occasionally, a differentiation between *BRCA1* and *BRCA2* carriers is made with a trend to start later or decrease mammography frequency in *BRCA1* compared to *BRCA2* carriers, probably based on the possible higher likelihood of microcalcifications as a reflection of in situ carcinoma in ER-positive breast cancers which are enriched in *BRCA2* [42, 45]. However, this differential correlation with in situ carcinoma has not been confirmed [78]. The age of onset of mammographic surveillance varies significantly between the different guidelines as described above. There seems to be a potential of adding digital breast tomosynthesis to the imaging surveillance for breast cancer in some women given the higher sensitivity and specificity compared to routine mammogram and possibly decreasing false positive findings of standard mammography, and some guidelines already describe this option [34, 37]. Individualization of starting age based on family history is recommended in the majority of guidelines. In the concept of shared decision making, patient preference is a very important consideration in the discussion of breast cancer surveillance strategies and risk-reducing options. There are still a lot of open questions regarding optimal breast cancer screening in *BRCA* carriers, e.g., recommended surveillance when MRI is not possible/unavailable, optimal age to discontinue surveillance, value of ultrasonography, and value of alternating versus concomitant imaging when 2 modalities are combined. These and other questions stress the importance of ongoing and future studies.

In contrast to RRSO, there is no evidence that screening for ovarian cancer in *BRCA* carriers reduces mortality. RRSO should be recommended for all *BRCA* mutation carriers, with important differences in age recommendations for RRSO between different guidelines. Some guidelines consider screening for ovarian cancer in people refusing RRSO. Others also consider surveillance in *BRCA* carriers before RRSO is performed. If this is considered, it is of utmost importance that patients are informed that there is no proven benefit of screening with serial CA125 measurements and transvaginal ultrasonography.

All the four guidelines that covered pancreatic carcinoma considered screening only in the presence of a positive familial history and after proper counseling of advantages and disadvantages of pancreatic cancer screening. There is no consensus as to whether it should be proposed to *BRCA2* carriers only or both, or about the screening modality or the age when screening should start. Because it is unknown if pancreatic cancer screening impacts overall survival, it is preferred to perform pancreatic cancer screening in the context of clinical trials and in high-volume centers.

The link between *BRCA* mutation carriers and prostate cancer has been well established. Screening is recommended by the NCCN, SEOM, and BeSHG guidelines, while the ESMO guidelines consider it [36, 37, 46, 51]. Differentiation is made between *BRCA1* and *BRCA2* carriers based on the higher penetrance of prostate cancer in male *BRCA2* carriers [67]. Most guidelines recommend PSA and DRE as screening methods, but optimal duration is not properly addressed.

Because there is less evidence about an association between *BRCA* pathogenic variants and colorectal, endometrial, skin, and gastric cancer, the majority of guidelines do not recommend systematic screening. Raised awareness and careful incorporation of familial history to individualize primary and secondary prevention for these cancer types seem appropriate. Further investigation of these cancer risks in *BRCA* carriers and evaluation of surveillance methods in clinical trials are warranted.

## 5. Conclusion

There are major differences between available guidelines for cancer surveillance in germline *BRCA* mutation carriers. Optimal surveillance strategies depend on accurate age-specific cancer risk estimates, which are reliably estimated for breast and ovarian cancer but not for other *BRCA*-related cancers. Up-to-date national or international consensus guidelines are of utmost importance to harmonize counseling and proposed surveillance strategies for *BRCA1/2* carriers. Improving awareness of carriers and primary care physicians together with shared decision making is a key aspect of cancer surveillance in *BRCA* carriers. Possible benefits of screening and risk-reducing strategies should always be discussed in combination with possible risks and limitations of these surveillance strategies.

## Figures and Tables

**Table 1:** Overview of lifetime cancer risks in carriers of germline *BRCA1/2* (likely) pathogenic variants.

Type of malignancy	Lifetime risk of malignancy		
	General population (%)	*BRCA1* (%)	*BRCA2* (%)
Breast, female [10, 11]	12	72	69
Breast, male [11–13]	0.1	1.2	6.8–8.4
Ovarian [10, 11]	1-2	44	17
Pancreatic^‡^ [14–16]	0.5	1–3	2–7
Prostate^‡^ [11, 17, 18]	6 (by age 65)	8.6 (by age 65)	15 (by age 65)
Colorectal^‡^ [11, 19]	4-5	Possibly elevated	¥
Endometrial^‡^ [11, 20]	3	Possibly elevated	¥
Melanoma^‡^ [11, 21]	2-3	¥	Possibly elevated

^‡^Lifetime risks not estimated, extrapolated from odds ratios/standardized incidence ratios. ^¥^Insufficient or inconsistent data about possible association with increased risk.
